# Optimization of Alkali-Activated Municipal Slag Composite Performance by Substituting Varying Ratios of Fly Ash for Fine Aggregate

**DOI:** 10.3390/ma14216299

**Published:** 2021-10-22

**Authors:** Mahmoud Abo El-Wafa, Kimio Fukuzawa

**Affiliations:** 1 Department of Civil Engineering, Faculty of Engineering-Rabigh Branch, King, Abdulaziz University, Jeddah 21589, Saudi Arabia; 2 Department of Civil Engineering, Faculty of Engineering, Aswan University, Aswan 81542, Egypt; 3 Department of Urban and Civil Engineering, Ibaraki University, Hitachi 316-8511, Japan; fukuzawa@ss.iij4u.or.jp

**Keywords:** alkali-activated, municipal slag, fly ash, cementitious materials, microstructure analysis, steam curing

## Abstract

This study investigates the effect of varying ratios of fly ash as a partial replacement for fine aggregate on the performance of alkali-activated municipal slag composites. The strength and other properties of alkali-activated cementitious material (AACM) composites can be optimized by selecting the appropriate mix proportion. In this study, we used fly ash as a substitute for fine aggregate (FA/S) at varying ratios of 0.0, 5.0, 10.0, 15.0, 20.0, 25.0, and 30.0%, mixed with 50% water (W/SL), and 20% alkali activator (AL/SL) content instead of municipal slag (SL) as a core binder, cured in steam conditions. The effects of these substitutions on the initial mixing temperature, slump flow, compressive and splitting tensile strengths, and microstructure analysis of composites cured in steam conditions were investigated at 1, 7, 28, and 91 days. The evaluation of the experimental results revealed that increasing the ratio of fly ash substitution to fine aggregate by up to 20.0% led to a higher strength attributable to the composites, whereas when the extra substitution ratio of FA/S ranged from 25.0–30.0%, significant decreases in strength were observed. The composites’ strengths were estimated using the ACI 209 and ACI 318 design equations and compared to the measured strengths.

## 1. Introduction

The construction sector is one of the main global marketplaces for supplementary cemented materials (SCMs). In the cement system, additional cemented materials (SCMs) can be added as components of concrete. These SCMs may be added to concrete as partial replacements for Portland cement, or they may be supplemented with cement, depending on the characteristics of the materials used and their effects on concrete [1,2]. SCMs can affects fresh or plastic concrete and can modify the properties of hardened concrete [3,4]. This is due to the SCMs’ prospected ability to improve the characteristics of concrete through their filler effect. The adoption of most of the SCMs that are produced from the by-product materials by the concrete industry might help to effectively dispose of these materials [5,6]. Moreover, the utilization of SCMs in the form of mineral admixtures serves as a substitute for cement, which might contribute towards preserving the non-renewable resources that are required for cement production, thereby potentially making construction materials more sustainable [7,8,9,10].

The advancements in the field of new materials recognized as alkali-activated cementitious materials (AACMs) that can be used as substitutes for Portland cement (PC), and their implications, have been the subject of rigorous study and investigation by the scientific community [11,12,13]. From the perspective of sustainable development, it has become an important issue to reduce the use Portland cement [14,15,16,17,18]. Wide-ranging inquiries into alternative mineral resources that could replace ordinary cement have recently shown that greenhouse gas emissions from cement furnaces have reduced [19,20,21,22,23]. Alkaline cementitious materials are a type of cementitious ingredient prepared by alkali-activator and latent hydraulic and cementitious materials that have been settled for more than 70 years [24,25,26,27,28]. Furthermore, the preparation of these materials falls within the domain of sustainability, and investigations into these kinds of materials have steadily increased on a global scale. Alkali-slag cement and fly ash are the two core divisions of alkaline cementitious materials and are classified as new alkali-activated cementitious materials (AACMs) [29,30,31]. They are the most recent types of AACM to be commercially introduced into the composites in the concrete industry. These AACMs are produced by blending municipal or industrial waste ingredients, such as slag cement (SL) or fly ash (FA), with alkaline-activating materials (AL). They generally demonstrate outstanding performance, but at the same time, each one of them has its own individual performance. Currently, additional intense investigation is required to fully comprehend the art of blending the two types of ingredients, complementing their individual performances, for the production of AACMs. These AACMs are favored for their greater durability and small environmental impact. Currently, due to enhanced access to AACMs, concrete producers are examining the possibilities and effects of combining two or more of these ingredients to enhance composites’ performance. The composition of these AACMs continues to be the subject of considerable debate in scientific research and depends on the physico-chemical nature of the raw materials, the quantity of activators, and the cured conditions. Alkali-activated slag mixtures usually exhibit greater mechanical properties. However, in alkali-activated slag mixtures, greater drying shrinkage has been detected than in similar OPC mixtures. On the other hand, fly ash-based alkali-activated mixtures typically manifest slower setting and increases in strength. The high content of glassy phase and reactive silica, along with its low iron oxide and calcium oxide content, affect the performance of alkaline-activated fly ash. Gharzounia et al. [32] observed that the strength increased by lowering the water-glass module from 1.64 to 1.0. It was found that adding a small amount of hydrated lime to sodium hydroxide, NaOH fly ash/slag combinations, and sodium silicate considerably enhanced their early-age strength [33]. The strength and hydration efficiency of NaOH-activated fly ash/slag pastes were studied in [34]. They found that the compressive strength of the blend with 50% fly ash/50% slag, activated with 10 M NaOH solution, was over 50 MPa at 28 days of age at 25 °C. The fly ash/slag ratio was the most significant issue in strength development. The effect of activating solutions (silicate solutions) on the mechanical and microstructural properties of alkali-activated fly ash geopolymers was investigated [35]. The alkali activators included aqueous solutions of Ca(OH)_2_, NaOH, NaOH+Na_2_CO_3_, KOH, and sodium silicate in various concentrations (water glass). They found that sodium silicate produced the greatest compressive strength. The atomic Si/Al ratio in the reaction products increased in tandem with the increase in sodium silicate SiO_2_/Na_2_O mass ratio. Again, the fly ash/slag ratio was the most significant issue in strength development [36,37,38,39,40].

In numerous cases, ground granulated blast furnace slag-Alkali activated cement or fly ash-based polymer cement was examined for making cement-less composites as the core binder. Dai et al. [36] considered the effects of the water-to-binder (w/b) ratio on the fresh state properties of alkali-activated cement (AAC) pastes using 50% type F fly ash (FA) and 50% ground-granulated blast furnace slag (GGBFS). They found that GGBFS provided the main contribution to the increase of storage modulus in the early stage of a hybrid mixture of GGBFS and FA. They also found that the w/b ratio of the mixture influenced the viscoelastic responses of GGBFS-FA mixtures. Lau et al. [37] successfully developed geopolymers with calcium content by combining ground-granular blast-furnace slag (GGBFS) with fly ash (FA) blended geopolymers. It was observed that at 40–50% FA replacement with GGBFS, the blended geopolymers achieved the majority of their 28-day compressive strength at 7 days. Zhang et al. [38] presented a thorough experimental investigation into the fracture properties of hardened alkali-activated slag/fly ash (AASF) pastes in relation to microstructure formation and reaction product composition. They discovered that increasing the slag content in AASF paste resulted in superior mechanical properties, such as compressive strength, elastic modulus, and fracture toughness.

Comparatively few investigations have been conducted up to now on the usage of blended slag cement with fly ash activated in alkali as an alternative to Portland cement [39,40]. M. A. Wafa and K. Fukuzawa [39] conducted an extensive experimental investigation into the simultaneous effect of alkali activator and water/slag cement ratios on composite properties through the full replacement of Portland cement at various ages under steam curing conditions. It was observed that the inclusion of a higher concentration ratio of alkali activator/slag cement (AL/SL) and a lower ratio of water/slag cement (W/SL) revealed a higher compressive strength of slag mortar composites under steam curing conditions for both early and late ages.

M.A. Wafa and K. Fukuzawa [40] conducted a thorough experimental investigation into the effects of different activator-to-main-binder ratios on the workability and early-age strength of alkali-activated municipal slag-fly ash-based geopolymer mortar under different curing conditions, such as steam curing (sc), water curing (wc), and air curing (ac). It was observed that the curing regimes as well as the activator content in the main binder are two significant factors influencing the performance evaluation of fresh mixtures and the early-age strength of geopolymer mortar.

However, there are still numerous challenges to be tackled. Therefore, this new study focused on producing slag cement as a new engineering material that could be used as a complete substitute for Portland cement, combined with fly ash as a partial substitution for fine aggregate, to create economically attractive performance mixtures and allow the concrete industry to become more sustainable. The aim of this study was to determine the most favorable mixture design for improving the properties of AACM composites with an acceptable workability and higher strength development, using a microstructure analysis and curing in steam at different ages, as well obtaining more information for determining the correct dosage rate for the composites by achieving the desired effect. Slag cement (SL) and fly ash (FA) have the potential to be used in new, sustainable methods of engineering cementitious materials and to provide the concrete construction industry with an increased capacity to meet more and more demanding requirements for the sustainability of construction materials. Consequently, it is important to develop procedures for designing and manufacturing alkali-activated cementitious materials (AACMs) by using high volumes of fly ash blended with slag cement. The steam curing of concrete has the advantage of accelerating the hydration reactions of cement. Therefore, the material develops compressive strength and reduces its permeability in a shorter time compared with standard curing under ambient conditions. 

The specific reason for steam-curing concrete is to increase the rate of strength gain and thus the rate of utilization of concrete production by ensuring that the concrete rapidly reaches a sufficient strength to allow early demolding or, in the case of prestressed concrete members, to enable the concrete to be stressed as soon as possible after casting. Furthermore, in the case of precast concrete construction, this is a requirement in some projects that must be completed quickly, especially in urban areas. The ACI 209 [41] and ACI 318 [42] design equations were used to estimate the compressive and split tensile strengths of the AACMs composites.

## 2. Experimental Details

### 2.1. Materials

Alkali-activated cementitious materials’ (AACMs) composites are considered among the promising and growing new materials used as cement substitutes. They are currently closely studied and investigated by the scientific community. Municipal slag (SL) and fly ash (FA) blended with alkali-activated (AL) materials are classified as new alkali-activated cementitious material (AACM) composites. Municipal slag (SL) made for municipal activities was used as the main binder for complete replacement of Portland cement. The fly ash (FA) class “F” was produced from coal burning by-products in power plants and was used in this study as a partial substitution to fine aggregate (S). Table 1 sets out the chemical components and physical properties of municipal slag (SL) and fly ash (FA).

Equations (1) and (2) define the basicity index for municipal slag (*k_b_*) and the hydration module (*HM*) with standard values of *k_b_* ≥ 1.0, *HM* > 1.4, and 1.3 ≤ *CaO*/*SiO*_2_ (C/S) ≤ 1.4 [43,44,45,46]. It was suggested that the municipal slag hydration module (*HM*) be used to exceed 1.4.

(1) $$k_{b}=\frac{CaO+MgO}{SiO_{2}+Al_{2}O_{3}}$$

(2) $$HM=\frac{CaO+MgO+Al_{2}O_{3}}{SiO_{2}}$$

As a result, the municipal slag basicity index (*k_b_*) = 1.0, hydration modulus (*HM*) = 1.8, and *CaO*/*SiO*_2_ (C/S) = 1.33.

Fly ash is graded as class “F” by ASTM C618-78 [47]. It exhibits pozzolanic activity and its silica, alumina, and iron oxide contents form at least 70% of its total mass, with a small percentage of calcium oxide (*CaO*) content not exceeding 10% of the total mass. White minute metasilicate sodium particles (Na_2_SiO_3_) were used as an alkali-activator (AL). The proportion of “SiO_2_” silicon dioxide to Na_2_O sodium oxide was 1.0 (SiO_2_ = 50.0%, and Na_2_O = 50.0%). Crushed sandstone was used as fine aggregate (S) in this study with a specific gravity of 2.58 and a fineness modulus of 2.83.

### 2.2. Mix Proportions

The proportions studied for the mixtures of the alkaline-activated cementitious materials (AACMs) are given in Table 2. All the composite mixtures of the AACMs were prepared at room temperature at 20 ± 1 °C and 65 ± 5% RH, then cured in steam. The parameters studied were municipal slag (SL) with a constant quantity as the core binder for the full replacement of Portland cement, combined with fly ash (FA) for the partial replacement of fine aggregate (S) by weight (FA/S: 0.0, 5.0, 10.0, 15.0, 20.0, 25.0 and 30%). An alkali activator/municipal slag (AL/SL) ratio of 20.0% and a water/municipal slag (W/SL) ratio of 50.0% were used and kept the same for all the mixes in order to create an economically desirable performance with complementary benefits and the possibility of sustainable development. The mixtures of AACM composites were selected according to their ingredients’ core variables in the mix. The mix designation (AACM-20) shown in Table 2 indicates that the AACM composite contained 20.0% of fly ash (FA) as a partial replacement for fine aggregate (S) (FA/S: 20.0 percent).

### 2.3. Sample Preparation and Test Methods

All the composite mixtures of AACM were set at room temperature, around 20 ± 1 °C, with an RH of around 65 ± 5%. All the raw ingredients (SL, FA, AL, and S) were mixed in a 90 s pan mixer. Water (W) was added, and a further 90 s of continuous mixing was performed to create the AACM composites. The AACM composites were investigated by conducting some tests to estimate the most favorable mixture designs for improving the property mixtures with an acceptable workability and higher strength development at different ages and cured in steam conditions, as well as to obtain information for determining the correct dosage rate of the composites by achieving the desired effect. The fresh properties tested included the initial mixing temperature and slump flow to verify the workability of the AACM composites. The initial mixing temperature and slump flow of the AACM composites were recorded directly after mixing. The JIS A 1150 (JSA 2014) [48] recommends the slump flow test for testing the workability of AACM composites through a flow table. The properties tested included compressive strength JIS A1108 (JSA 2006a) [49] and split tensile strength JIS A1113 (JSA 2006c) [50] cured in steam at various ages. The electron microscopic scanning (SEM) technique was used to examine the microstructure of the AACM composites cured in steam at various ages. All the sample molds were performed at 20 ± 1 °C, room temperature, with 65 ± 5% RH and covered with plastic sheets. Subsequently, after 1 h of casting, all the samples’ molds were placed inside a steam chamber to start the steam curing procedure.

### 2.4. Samples Curing Procedure

**Figure 1:**
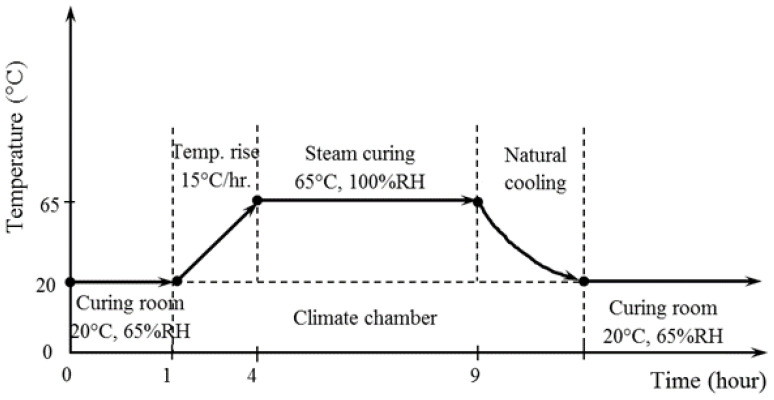
Schematic description of the steam curing process.

Figure 1 shows a schematic representation of the AACM composite steam curing process. All the sample molds were placed in a steam chamber after 1 h of casting to begin the steam curing procedure. After that, the steam chamber temperature was increased at a ratio of 15 °C/h from 20 °C to 65 °C and maintained at 65 °C for an extra 5 h, then cooled down to 20 °C in normal mode.

All the molds were removed one day after casting and maintained at 20 ± 1 °C room temperature with 65 ± 5% RH until the day of testing. In order to explain the typical compressive and splitting tensile strength cured in steam, three samples of individual mixtures were examined at ages 1, 7, 28 and 91 days; hence, the strength development could be observed over time. The microstructure analysis of the AACM composites was conducted by using the technique of scanning electron microscopy (SEM) with ratios of (FA/S: 20%, AL/SL: 20.0% and W/SL: 50.0%) at 1, 28, and 91 days of steam curing.

## 3. Results and Discussion

### 3.1. Initial Mixing Temperature of AACMs Composites

The initial mixing temperature is one of the main issues that can directly affect the properties of fresh and hardened AACM composites in the form of increased water demand, accelerated slump loss, reduced setting time, improved plastic shrinkage pattern, decreased strength, and reduced durability. The appropriate temperature is therefore an integral factor in any composite quality control scheme. Table 3 provides a summary of test results of the initial mixing temperature of the fresh AACM composites, whose proportion of alkali activator and water to municipal slag as the core binder was constant (AL/SL: 20% & W/SL: 50%), as well as the results of various replacement ratios of fly ash to fine aggregate as FA/S ( 0.0, 5.0, 10.0, 15.0, 20.0, 25.0, and 30.0%).

**Figure 2:**
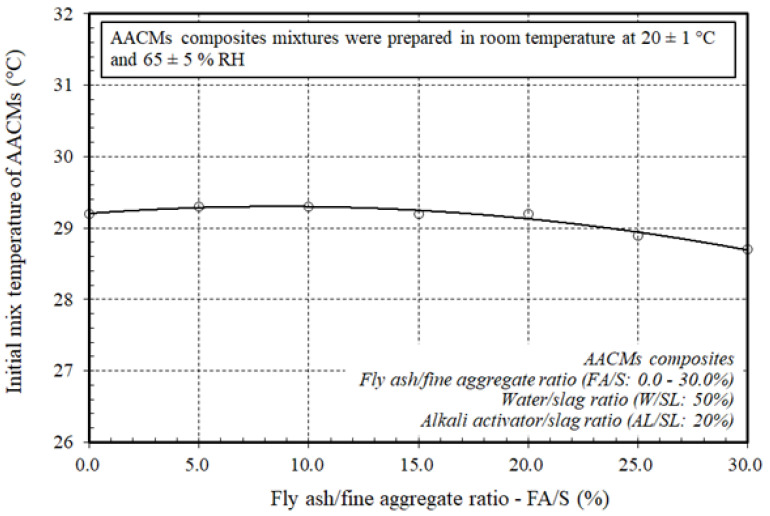
Effect of varying ratios of fly ash to fine aggregate on the initial mixing temperature of the AACMs composites.

The effect on the initial mixing temperature of the AACM composites of the varying replacement ratios of fly ash to fine aggregate as FA/S is shown in the Figure 2. From the results obtained, different effects on the initial mixing temperature of the AACM composites, depending on the differing replacement ratios between fly ash and fine aggregate (FA/S) used, were noted. The initial mixing temperature values were 29.2–29.3 °C with varying replacement ratios of FA/S ranging from 0.0–20.0%, whereas the initial mixing temperature values were 28.9–28.7 °C with higher replacement ratios of FA/S ranging from 25.0–30.0%, respectively.

The reduction in the rate of temperature produced and, hence, the initial mixing temperature of the composites of the AACMs, led to the of use fly ash class “F”, which exhibited a pozzolanic reaction, and the silica, alumina, and iron oxide content reached 70% of the total mass with a small percentage of calcium oxide (*CaO*) content, less than 10%, resulting in decreased heat generation and delayed setting times. Consequently, AACM composites with a high fly ash substitution to fine aggregate (FA/S) ratio are particularly suitable for minimizing autogenous temperature rises directly affecting slump loss, increasing setting time and reducing the strengths of AACMs composites. Therefore, when the higher fly ash substitution to fine aggregate (FA/S) ratio was used, it produced lower initial mixing temperatures. This performance was significant because the impact of the hydration procedure on the setting time of the composite AACMs was delayed.

### 3.2. Slump Flow of AACM Composites

Slump value is one of the important characteristics of the workability of fresh concrete properties. The slump flow experiment was approved to check the workability of the AACM composites. The JIS A 1150 (JSA 2014) [48] was used to control the workability of the fresh AACMs composites by measuring the slump flow immediately after mixing. Table 3 presents a summary of the initial slump flow values of the fresh AACM composites. These composites of AACMs had a constant ratio of alkaline activator and water to municipal slag as the core binder (AL/SL: 20% & W/SL: 50%), and varying replacement ratios of fly ash to fine aggregate (FA/S): 0.0, 5.0, 10.0, 15.0, 20.0, 25.0, and 30.0%. The difference between the workability of the fresh AACMs composites and the fly ash was solely due to the ratio of fly ash substitution to fine aggregate (FA/S); all the other material proportions were kept constant.

**Figure 3:**
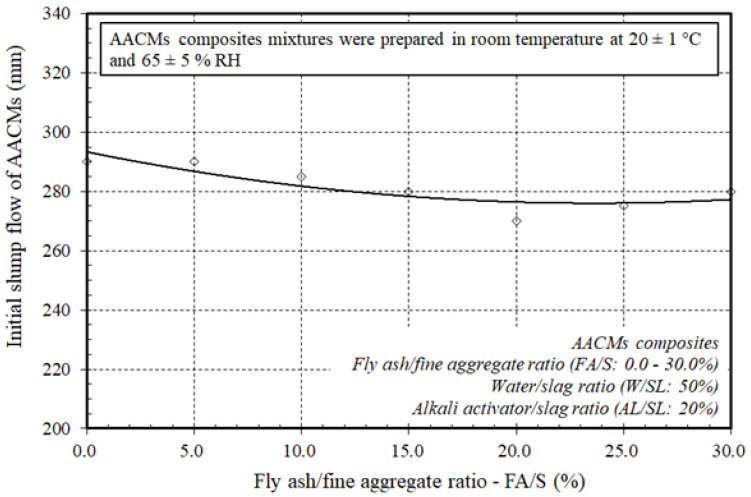
Effect of varying ratios of fly ash to fine aggregate on the initial slump flow of the AACMs composites.

Figure 3 shows the effect of varying the ratio of fly ash substitution to fine aggregate (FA/S) on the initial slump flow of the AACM composites. The initial slump flow values were 290–270 mm with varying replacement ratios of FA/S ranging from 0.0–20.0%, whereas the initial slump flow values were 275–280 mm with higher replacement ratios of FA/S ranging from 25.0–30.0%, respectively. It was inferred from the obtained results that the workability reduced as the ratio of fly ash substitution to fine aggregate increased up to 20.0%. This was due to the increase in the quantity of silicon dioxide in line with increase in the ratio of fly ash substitution ratio to fine aggregate (FA/S) ranging from 0.0–20.0%, which made the mixture very sticky and matched with the values of the initial mixing temperature generated with almost constant values of about 29.2–29.3 °C. However, up to the replacement ratio of fly ash to fine aggregate (FA/S: 20%), the mixture was still workable. With additional replacement ratios of FA/S ranging between 25.0–30.0%, it was detected that the workability increased, which matched more with the lower values of the initial mixing temperature generated at about 28.9–28.7 °C than those generated at 29.2–29.3 °C with a replacement ratio of (FA/S) ranging from 0.0–20.0%. Therefore, the AACM composites with a high ratio of fly ash substitution to fine aggregate (FA/S) were particularly suitable for minimizing autogenous temperature rises, which directly slowed down the slump loss and increased the setting time.

### 3.3. Compressive Strength Development of AACM Composites

The hardened properties of the AACM composites were examined by conducting a compressive strength test to evaluate the most favorable mixture designs for improving the property mixtures with higher compressive strength development cured in steam at different ages, as well as to obtain more information for determining the correct dosage rate of the composites by achieving the desired effect. To define the typical compressive strength of JIS A1108 (JSA 2006a) [49] cured in steam, three samples of individual mixtures, aged 1, 7, 28, and 91 days, were inspected; hence, strength development could be observed over time.

**Figure 4:**
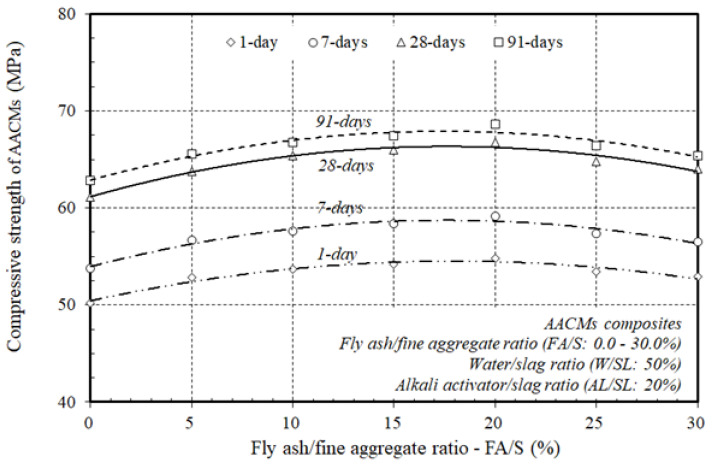
Effect of varying ratios of fly ash to fine aggregate on the compressive strength of the AACM composites.

Figure 4 displays the varying ratios of fly ash substitution to fine aggregate (FA/S), 0.0, 5.0, 10.0, 15.0, 20.0, 25.0, and 30%, and their impact on the production of the compressive strength of the AACM composites with a constant ratio of alkaline activator and water to municipal slag as the core binder (AL/SL: 20% & W/SL: 50%), at 1, 7, 28, and 91 days. The discrepancy between the gains in compressive strength of the AACM composite hardened materials and the fly ash was entirely related to the ratio of the fly ash substitution to fine aggregate (FA/S); all the other material ratios were kept constant. When the ratio of the fly ash substitution to the fine aggregate (FA/S) rose to 20.0%, the production of compressive strength increased, and with an increase in the ratio of fly ash substitution to fine aggregate (FA/S) from 25.0 to 30.0%, the compressive strength development declined at 1, 7, 28, and 91 days. In the composite matrix, the effect of the rise in the replacement ratio (FA/S) up to 20.0% on compressive strength growth was more distinct, as it strengthened the microstructure of the composites by creating a denser binder and matched the workability results obtained by increasing the replacement ratio (FA/S) to 20.0%, rendering the mixture very sticky. Nevertheless, as the FA/S replacement ratios ranged from 25.0–30.0% in the core binder, the compressive strength decreased considerably. The decline in the compressive strength was due to the lower rate of heat produced; hence the initial mixing temperature of the AACM composites led to the use of fly ash class “F”, which exhibited a pozzolanic reaction, resulting in decreased heat generation and delayed setting times while increasing the workability of the AACMs composites. Hence, the compressive strength decreased. Therefore, the higher replacement ratios of (FA/S) ranged from 25.0–30.0% had a potentially adverse influence on the compressive strength due to the lower rate of heat produced.

However, the higher replacement ratios directly delayed the setting time and slowed down the slump loss with reasonable reductions in the compressive strength of the AACM composites, which is suitable in constructions such as dams, bridge piers and abutments, and large concrete footings. The results clearly indicated that the optimum replacement ratios of (FA/S) on the compressive strength of the composite AACMs were about 20.0%. This was directly linked to the enhancement of the microstructure of the matrix by the formation of a denser binder and the higher rate of heat produced and, hence, to the higher initial mixing temperature generated. Consequently, as the substitution ratio (FA/S) rose to 20.0%, the compressive strength of the AACM composites became more beneficial and acceptable for practical use than that obtained with the higher replacement ratios of (FA/S) with reasonable reductions in the compressive strength, which is suitable in some engineering applications, such as dams, bridge piers and abutments, and large concrete footings.

**Figure 5:**
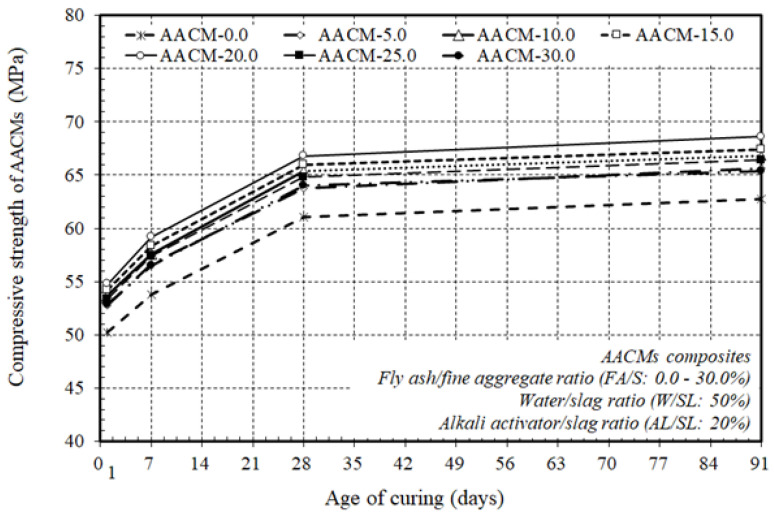
Compressive strength growth of AACM composites cured in steam at various ages.

Figure 5 plots the compressive strength growth of the AACM composites at 1, 7, 28, and 91 days with varying ratios of fly ash substitution to fine aggregate (FA/S), 0.0, 5.0, 10.0, 15.0, 20.0, 25.0, and 30.0% cured in steam.

The general trend of the growth of the AACM composites’ compressive strength at 1, 7, 28, and 91 days showed a similar pattern with differing replacement ratios of (FA/S) cured in steam. At 1, 7, and 91 days of steam curing, the compressive strength improved to about 82, 88, and 102% of the 28-day compressive strength of the AACM composites, respectively. The compressive strength growth of the AACM composites improved with time, and the strength development trends at 1, 7, and 91 days were identical to those at 28 days. Figure 5 indicates that when the core binder combined a higher replacement ratio of (FA/S) up to 20.0%, the compressive strength improved considerably, but with the extra replacement ratios of (FA/S) ranging from 25.0–30.0%, the compressive strength decreased with a lower rate and with reasonable reductions. The decline in strength was due to the delaying of the setting time and the slowdown of the slump loss of the AACM composites.

Therefore, the extra replacement ratios of (FA/S) ranging from 25.0–30.0% possibly had an undesirable influence on the AACM composites due to a lack of accessibility for hydration.

### 3.4. Split Tensile Strength Development of AACMs Composites

**Figure 6:**
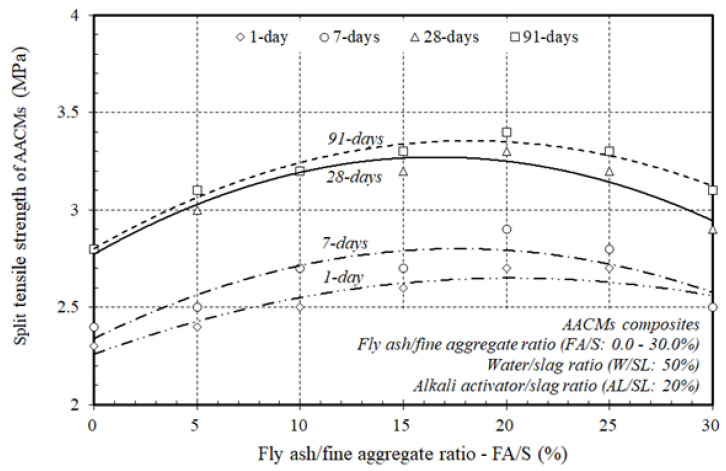
Effect of varying ratios of fly ash to fine aggregate on the split tensile strength of the AACM composites.

To define the typical split tensile strength of JIS A1113 (JSA 2006c) [50] cured in steam, three samples of individual mixtures, aged 1, 7, 28, and 91 days, were inspected; hence, the split tensile strength development could be observed over time. Figure 6 displays the split tensile strength development of the AACM composites cured in steam with varying ratios of fly ash substitution to fine aggregate (FA/S), 0.0, 5.0, 10.0, 15.0, 20.0, 25.0 and 30.0%, and constant proportions of the alkali activator and water to municipal slag as the core binder (AL/SL: 20% & W/SL: 50%) at 1, 7, 28, and 91 days. The development of split tensile strength increased as the fly ash substitution ratio to fine aggregate (FA/S) increased up to 20.0%, and as the ratio of fly ash substitution to fine aggregate (FA/S) rose from 25.0 to 30.0%, the development of split tensile strength decreased at 1, 7, 28, and 91 days. The replacement ratio of (FA/S) therefore had the highest result on the split tensile strength up to 20.0% of the replacement ratio of (FA/S). This trend was similar to the pattern of increase in compressive strength. The findings therefore state that the split tensile strength of AACM composites is positively influenced by an improvement in the replacement ratio of (FA/S) up to 20.0%, which is close to the previously discussed production of compressive strength.

**Figure 7:**
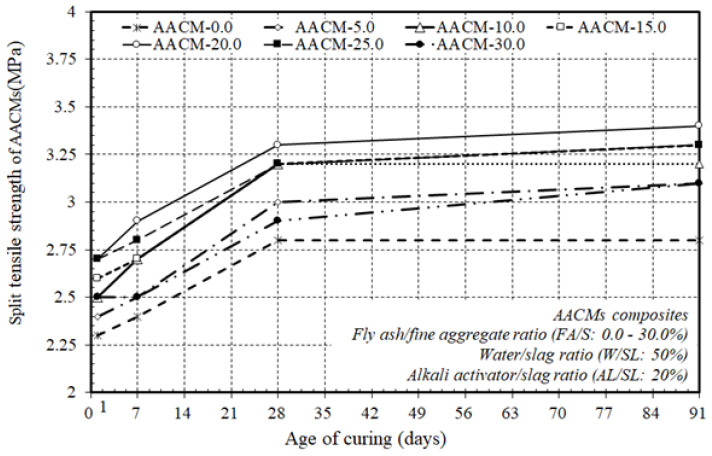
Split tensile strength growth of AACM composites cured in steam at various ages.

Figure 7 exhibits the split tensile strength of the composite AACMs cured in steam at 1, 7, 28, and 91 days. The improvement in the split tensile strength increased with time in the AACM composites. The split tensile strength of AACM composites improved rapidly at 1 and 7 days of age to around 82 and 86% of the 28-day steam-cured split tensile strength, while at the age of 91 days it increased gradually to about 102% of the 28-day split tensile strength. The trend towards the increase in split tensile strength at 1, 7, and 91 days was close to that observed at 28 days.

Figure 7 also demonstrates that the split tensile strength of the AACM composites improved when increasing the replacement ratio of (FA/S) up to 20.0% in the AACM composites cured in steam conditions, but with the extra replacement ratios of (FA/S) ranging from 25.0–30.0%, the splitting tensile strength decreased at a lower rate and with reasonable reductions. This trend was close to that of the compressive strength development of the AACM composites, but at a smaller level. The improvement in the splitting strength of the AACM composites was achieved by increasing the replacement ratio of (FA/S) in the AACM composites up to 20.0%, which was identical to the development of the compressive strength discussed earlier.

The results indicated that the replacement ratio of (FA/S) up to 20.0% resulted in the highest split tensile strength. This was because the microstructure of the matrix was affected by the formation of a denser binder and the higher rate of heat produced and, hence, the higher initial mixing temperature generated, which was produced by the chemical reactions. This effect shows that the application of a replacement ratio of (FA/S) up to 20.0% is a more effective way to enhance the splitting tensile strength of the AACM composites than the application of extra replacement ratios of (FA/S) ranging from 25.0–30.0%. The results of the split tensile strength were identical to those of the compressive strength of the AACM composites cured in steam.

### 3.5. Microstructure Analysis of AACMs Composites

**Figure 8:**
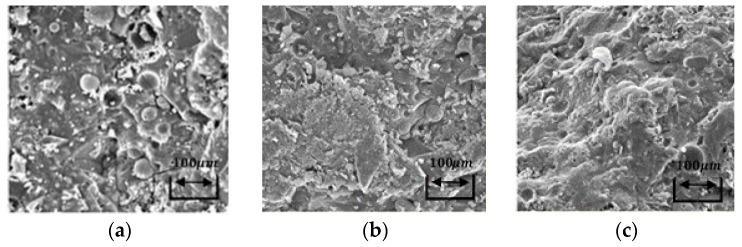
SEM micrographs of AACM composites cured in steam at: (**a**) 1 day; (**b**) 28 days; (**c**) 91 days.

Scanning electron microscopy at 1, 28, and 91 days of age studied the microstructure of AACM composites cured in steam with a 20% ratio of fly ash replacement to fine aggregate (FA/S: 20%), 50% water to municipal slag as the core binder (W/SL: 50%), and 20% alkali activator to municipal slag as the core binder (AL/SL: 20%), respectively (see Figure 8a–c). Figure 8a–c demonstrates that the fly ash particles had a smooth spherical form, but the municipal slag particles demonstrated variable angular form sizes. When the alkaline activator (Na_2_SiO_3_) was used, the N-A-S-H gel did not form alongside the C-S-H gel, and the majority of the urban slag particles were dispersed. Due to the small quantity of silica present in the resin, some incompletely disbanded slag particles did not form the C-S-H gel.

Homogeneously scattered microcracks formed in the exterior surface of the composites. Nevertheless, smaller numbers of microcracks in the denser microstructure were found. This suggested that the C-S-H gel did not contain all the slag particles in the matrix of AACM composites. The microstructure was highly compact, with fewer unreacted slag particles. There were a number of condensed microcracks, but the most extensive cracks formed at the C-S-H boundary.

Figure 8a–c reveals that the fly ash created a large amount of hollow spheres. Roughly unreacted fly ash particles were also observed. The separate small holes were formed in the matrix, when the hollow circular particles were slightly dispersed. These unreacted particles were produced in hollow bores, which could have been the product of the gap by leaving behind the dispersed particles of fly ash. The spherical morphology is useful for the matrix of AACM composites, as it transfers fitted workability to binder ratios at lower water contents. The microcracks were further scattered across the surface and the boundaries of the unreacted or more affected particles (see Figure 8a–c).

Moreover, the microstructure of the AACM composites featured an extra condensed matrix. The water-to-core-binding ratio affected the hole sizes and the permeability of the composites, which directly controlled the strength of the AACM composites. Upon exposure to steam curing, all the composites demonstrated thick matrices and rough textures, as well as a large amount of micropores (see Figure 8a–c).

The initiation of hole formation refers to the Si:Al composite ratio of the AACMs. The Si content in the composites of the AACMs lost the initiation temperature of the production hole as a result of the removal of water hydration when the bubbles were created, resulting in the formation of close bubbled components. In addition, unreacted or even reacted particles remained apparent. The microstructure of the AACM composites therefore revealed a microstructure with dense particles collected with excessive integrated holes, as the test samples with Si:Al percentages were uniform at 1.65, with porosity distributed in tiny holes.

An earlier inspection of the microstructure of the AACM composites demonstrated that the growth of the microstructure as the silicon volume increased within the small compositional zone was rapid and incessant. The proportion of Si to Al impacted the hole-size distribution of the AACM composites. The hole-size distributions of the AACM composites tended to be smaller as the Si:Al ratio increased.

Thus, the AACM composites cured in steam appeared to be denser and less porous and, explaining their current established strengths. Figure 8b indicates a considerable improvement of the steady hole size on the 28-day composite surfaces that might have resulted from a partial failure leading to a comparative increase in the progression of the crack size in the composite matrices of the AACMs.

## 4. Predictions of AACM Composite Strength

The ACI 209 Code (ACI 1997) [41] estimated the compressive strength of AACMs composites cured in steam, and the split tensile strength was calculated by the ACI 318 Code (ACI 2011) [42]. It used the following equation for determining compressive strength (ACI 1997) [41]:

(3) $$f_{c}^{’}\left(t\right)=\frac{t}{a+\beta t}\;{\left(f_{c}^{’}\right)}_{28}$$

where $f_{c}^{’}\left(t\right)$ = compressive strength; a and β = coefficients depending upon the kind of cement used and the condition of its cure; t = concrete age in days; and ${\left(f_{c}^{’}\right)}_{28}$ = compressive strength of 28-day. The standard ACI 209 states that a and β are 0.7 and 0.98 for the steam-cured OPC concrete, respectively. The basic ACI 318 [42] addresses Equation (4) for calculating the split tensile strength from the average compressive strength of 28 days. In most cases, Equation (4) is considered sufficient to calculate the split tensile strength as the average value of the specific unit weight of concrete:

(4) ft‘=gt[w(fc‘)t]12

where $g_{t}\;$= 0.0069 average constant value; w = concrete unit weight in kg/m^3^—the mean unit weight of the AACMs composites used in this analysis was 2150 kg/m^3^; and $f_{c}^{’}\left(t\right)\;$= compressive strength at time (t). Therefore, the Equation (4) becomes:

(5) $$f_{t}^{‘}\;\left(MPa\right)=0.32\sqrt{{\left(f_{c}^{‘}\right)}_{t}}$$

Consequently, the split tensile strength $f_{t}^{‘}\;\left(MPa\right)\;$of the AACM composites was considered in accordance with ACI 318 (see Equation (5)).

**Figure 9:**
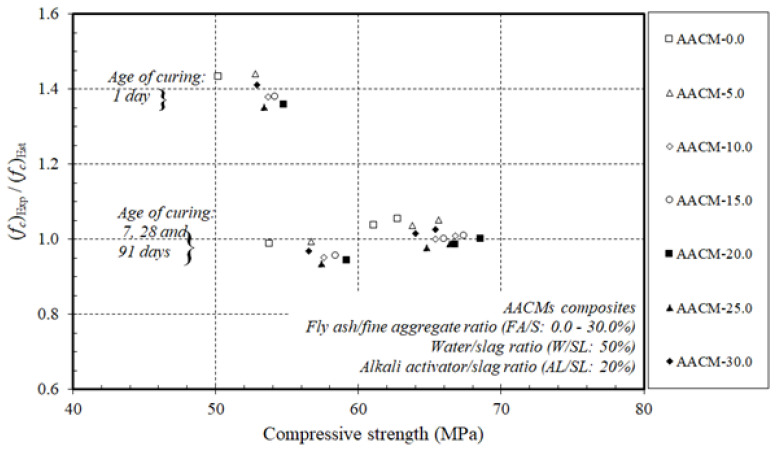
Ratio of experimental compressive strength to compressive strength estimated by ACI 209 of AACM composites cured in steam.

**Figure 10:**
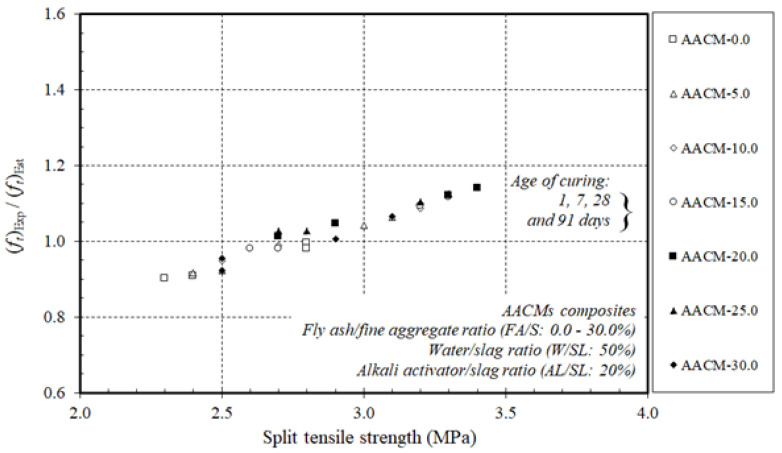
Ratio of experimental split tensile strength to split tensile strength estimated by ACI 318 of AACMs composites cured in steam.

Figure 9 and Figure 10 display the ratios of the experimental results to the estimated values by the design Equations of ACI 209 and ACI 318 for the strengths of the compressive $f_{c}^{‘}$ and the split tensile $f_{t}^{‘}$ of the AACM composites.

The experimental proportions of the AACM composites cured in steam were equivalent to those of the OPC concrete calculated by ACI 209 and ACI 318. For the 1-day duration, the ratio of the experimental to the predicted proportions was higher than for the 7-, 28-, and 91-day compressive strength of the AACM composites cured in steam.

In general, there is little information on the accuracy and validity of using ACI Equations designed for concrete on AACMs to estimate compressive and split tensile strengths. Yang et al. [51] recorded that AASC splitting tensile strengths fit well with the ACI code. Furthermore, Chi [52] reported that AASC splitting tensile strengths agreed well with the value predicted by ACI code 318. Moreover, Yang et al. [53] noted that when the alkali quality coefficient was greater, the measured compressive strength was always greater than the predictions obtained from the design equations specified in ACI 209. The measured compressive strength of the FA-based AA mortars, on the other hand, was higher than that predicted by ACI 209 at ages greater than 7 days. Furthermore, Fang et al. [54] stated that the existing ACI code equations for OPC concrete overestimated the values of splitting tensile strength, but mostly followed the same development trend as the compressive strength of AAFS concrete cured at ambient temperature.

As general recommendations, standards such as the ACI code can be applied to AACMs, but the results are conservative. They are, however, sufficient for predicting the properties of AACMs. Based on the results presented in Figure 9 and Figure 10, the ACI 209 and ACI 318 provided appropriate calculations of the compressive $f_{c}^{‘}$ and split tensile $f_{t}^{‘}$ strengths of AACM composites with corresponding experimental results.

## 5. Conclusions

The mixing effect of alkali-activated municipal slag as a core binder for the full replacement of Portland cement with 50% water content, 20% activator-content-to-binder ratios, and ratios of fly ash substitution to fine aggregate in the binder at 0.0, 5.0, 10.0, 15.0, 20.0, 25.0 and 30.0%, in order to introduce new alkali-activated cementitious material (AACM) composites, cured in steam at ages of 1, 7, 28, and 91 days, were examined experimentally. The effects of different replacement ratios of fly ash to fine aggregate on the initial mixing temperature, slump flow, compressive strength, split tensile strength, and microstructure analysis of AACM composites cured in steam at 1, 7, 28, and 91 days of age were observed. The optimization of the binding mechanism and the properties of the composites of the AACMs were assessed. Predictions of the strengths of the AACMs composites were also performed using ACI design codes. The following conclusions can be drawn from this analysis:

1. Sustainability and environmental issues are considered as a dual challenge. These were explored by combining municipal slag activated in alkali form with fly ash for the development of new cementitious materials as cement replacements in the core binde. These played a dynamic role in the production of AACM composites and had a major impact on the properties of both fresh and hardened composites.
2. The varying ratio of fly ash substitution to fine aggregate was a significant factor influencing the performance of the AACM composites. Hence, the most favorable mixture design was obtained for improving the properties of composites with an acceptable workability, higher strength development, microstructure analysis, and steam-curing at different ages.
3. The assessment of varying ratios of fly ash substitution to fine aggregate up to 20.0% suggested that the developed strengths were attributed to the AACM composites, which is achievable in order to produce the most favorable mixture designs. The extra replacement ratios of FA/S ranging from 25.0–30.0% were incorporated in the main binder; the strengths decreased significantly, a with potentially adverse influence on the strengths, but with reasonable reductions in the strengths of the AACM composites. This could make the composites suitable for constructions such as dams, bridge piers and abutments, and large concrete footings.
4. The microstructural performance of AACM composites cured in steam conditions had the greatest impact. These composites are applicable in prefabricated concrete construction and other engineering applications requiring greater strength.
5. The ACI 209 and ACI 318 provided appropriate estimations of the compressive$\;f_{c}^{‘}$, and split tensile $f_{t}^{‘}$ strengths of AACM composites with corresponding experimental results.

## Figures and Tables

**Table 1:** Municipal slag and fly ash: chemical components and physical properties.

Chemical Components and Physical Properties	Municipal Slag	Fly Ash
Calcium oxide, CaO (%)	43.1	6.3
Silicon dioxide, SiO_2_ (%)	32.5	57.6
Aluminum oxide, Al_2_O_3_ (%)	13.5	26.5
Magnesium oxide, MgO (%)	2.9	1.2
Ferric oxide, Fe_2_O_3_ (%)	2.7	4.2
Sodium oxide, Na_2_O (%)	1.8	0.5
Titanium dioxide, TiO_2_ (%)	1.3	1.9
Phosphorus pentoxide, P_2_O_5_ (%)	0.8	0.3
Loss on ignition, LOI (%)	1.4	1.5
Specific gravity (g/cm^3^)	2.80	2.14
Specific surface area (cm^2^/g)	3750	3630
Average particle size of D_50_ (μm)	6.48	18.35

**Table 2:** Mix proportions of alkaline-activated cementitious materials composites.

Mix Designation	Fly Ash/Fine Aggregate Ratio Fa/S (%)	Alkali/Binder Ratio AL/SL (%)	Water/Binder Ratio W/SL (%)	Mix Proportioning (kg/m^3^)				
				Binder (SL)	Water (W)	Alkali-Activator(AL)	Fly Ash (FA)	Fine Aggregate (S)
AACM-0	0.0	20	50	600	300	120	0	1200
AACM-5	5.0						58	1142
AACM-10	10.0						110	1090
AACM-15	15.0						157	1043
AACM-20	20.0						200	1000
AACM-25	25.0						240	960
AACM-30	30.0						277	923

**Table 3:** Fresh properties of alkaline-activated cementitious materials composites.

Mix Designation	Fly Ash/Fine Aggregate Ratio FA/S (%)	Alkali/Binder Ratio AL/SL (%)	Water/Binder Ratio W/SL (%)	Fresh Properties	
				Initial Mixing Temperature (°C)	Initial Slump Flow (mm)
AACM-0	0.0	20	50	29.20	290
AACM-5	5.0			29.30	290
AACM-10	10.0			29.30	285
AACM-15	15.0			29.20	280
AACM-20	20.0			29.20	270
AACM-25	25.0			28.90	275
AACM-30	30.0			28.70	280

Note: All mixtures of AACM composites were prepared at room temperature, 20 ± 1 °C, and 65 ± 5% RH.

## Data Availability

The data that support the findings of this study are available from the corresponding author, upon reasonable request.

## References

[1] Bashar I.I., Alengaram U.J., Jumaat M.Z., Islam A. (2016). Development of Sustainable Geopolymer Mortar using Industrial Waste Materials. Mater. Today Proc..

[2] Duxson P., Jimenez A.M.F., Provis J., Lukey G.C., Palomo A., Van Deventer J.S.J. (2006). Geopolymer technology: The current state of the art. J. Mater. Sci..

[3] Pacheco-Torgal F., Miraldo S., Labrincha J., de Brito J. (2012). An overview on concrete carbonation in the context of eco-efficient construction: Evaluation, use of SCMs and/or RAC. Constr. Build. Mater..

[4] Liu S., Zhang T., Guo Y., Wei J., Yu Q. (2018). Effects of SCMs particles on the compressive strength of micro-structurally designed cement paste: Inherent characteristic effect, particle size refinement effect, and hydration effect. Powder Technol..

[5] Chunlin L., Kunpeng Z., Depeng C. (2011). Possibility of Concrete Prepared with Steel Slag as Fine and Coarse Aggregates: A Preliminary Study. Procedia Eng..

[6] Hale W.M., Freyne S.F., Bush T.D., Russell B.W. (2008). Properties of concrete mixtures containing slag cement and fly ash for use in transportation structures. Constr. Build. Mater..

[7] Moreno-Juez J., Vegas I.J., Rojas M.F., de la Villa R.V., Guede-Vázquez E. (2020). Laboratory-scale study and semi-industrial validation of viability of inorganic CDW fine fractions as SCMs in blended cements. Constr. Build. Mater..

[8] Skibsted J., Snellings R. (2019). Reactivity of supplementary cementitious materials (SCMs) in cement blends. Cem. Concr. Res..

[9] Rahla K.M., Mateus R., Bragança L. (2019). Comparative sustainability assessment of binary blended concretes using Supplementary Cementitious Materials (SCMs) and Ordinary Portland Cement (OPC). J. Clean. Prod..

[10] Schöler A., Lothenbach B., Winnefeld F., Ben Haha M., Zajac M., Ludwig H.-M. (2017). Early hydration of SCM-blended Portland cements: A pore solution and isothermal calorimetry study. Cem. Concr. Res..

[11] Liu J., Hu L., Tang L., Ren J. (2020). Utilisation of municipal solid waste incinerator (MSWI) fly ash with metakaolin for preparation of alkali-activated cementitious material. J. Hazard. Mater..

[12] Singh J., Singh S.P. (2019). Development of Alkali-activated Cementitious Material using Copper Slag. Constr. Build. Mater..

[13] Zhang Q., Ji T., Yang Z., Wang C., Wu H.-C. (2019). Influence of different activators on microstructure and strength of alkali-activated nickel slag cementitious materials. Constr. Build. Mater..

[14] Cinquepalmi M.A., Mangialardi T., Panei L., Paolini A.E., Piga L. (2008). Reuse of cement-solidified municipal incinerator fly ash in cement mortars: Physico-mechanical and leaching characteristics. J. Hazard. Mater..

[15] Gartner E., Macphee D.E. (2011). A physico-chemical basis for novel cementitious binders. Cem. Concr. Res..

[16] Juenger M., Winnefeld F., Provis J., Ideker J. (2011). Advances in alternative cementitious binders. Cem. Concr. Res..

[17] Li C., Sun H., Li L. (2010). A review: The comparison between alkali-activated slag (Si+Ca) and metakaolin (Si+Al) cements. Cem. Concr. Res..

[18] Istuque D., Soriano L., Akasaki J., Melges J., Borrachero M., Monzó J., Payá J., Tashima M. (2019). Effect of sewage sludge ash on mechanical and microstructural properties of geopolymers based on metakaolin. Constr. Build. Mater..

[19] Ranjbar N., Mehrali M., Behnia A., Alengaram U.J., Jumaat M.Z. (2014). Compressive strength and microstructural analysis of fly ash/palm oil fuel ash based geopolymer mortar. Mater. Des..

[20] Manso J., Polanco J.A., Losañez M., Gonzalez J.J. (2006). Durability of concrete made with EAF slag as aggregate. Cem. Concr. Compos..

[21] Ozturk M., Karaaslan M., Akgol O., Sevim U.K. (2020). Mechanical and electromagnetic performance of cement based composites containing different replacement levels of ground granulated blast furnace slag, fly ash, silica fume and rice husk ash. Cem. Concr. Res..

[22] Troschinetz A.M., Mihelcic J.R. (2009). Sustainable recycling of municipal solid waste in developing countries. Waste Manag..

[23] Yusuf M.O., Johari M.A.M., Ahmad Z.A., Maslehuddin M. (2014). Evolution of alkaline activated ground blast furnace slag–ultrafine palm oil fuel ash based concrete. Mater. Des..

[24] Roy D.M. (1999). Alkali-activated cements Opportunities and challenges. Cem. Concr. Res..

[25] Kürklü G. (2016). The effect of high temperature on the design of blast furnace slag and coarse fly ash-based geopolymer mortar. Compos. Part B Eng..

[26] Singh B., Ishwarya G., Gupta M., Bhattacharyya S. (2015). Geopolymer concrete: A review of some recent developments. Constr. Build. Mater..

[27] Khater H., el Gawaad H.A. (2016). Characterization of alkali activated geopolymer mortar doped with MWCNT. Constr. Build. Mater..

[28] Singh N., Middendorf B. (2019). Geopolymers as an alternative to Portland cement: An overview. Constr. Build. Mater..

[29] Zhang G., Yang H., Ju C., Yang Y. (2020). Novel selection of environment-friendly cementitious materials for winter construction: Alkali-activated slag/Portland cement. J. Clean. Prod..

[30] Wu Y., Lu B., Bai T., Wang H., Du F., Zhang Y., Cai L., Jiang C., Wang W. (2019). Geopolymer, green alkali activated cementitious material: Synthesis, applications and challenges. Constr. Build. Mater..

[31] Luo X., Xu J., Bai E., Li W. (2012). Systematic study on the basic characteristics of alkali-activated slag-fly ash cementitious material system. Constr. Build. Mater..

[32] Gharzouni A., Vidal L., Essaidi N., Joussein E., Rossignol S. (2016). Recycling of geopolymer waste: Influence on geopolymer formation and mechanical properties. Mater. Des..

[33] Shi C., Jimenez A.M.F., Palomo A. (2011). New cements for the 21st century: The pursuit of an alternative to Portland cement. Cem. Concr. Res..

[34] Palomo A., Grutzeck M., Blanco-Varela M.T. (1999). Alkali-activated fly ashes: A cement for the future. Cem. Concr. Res..

[35] Komljenović M., Baščarević Z., Bradić V. (2010). Mechanical and microstructural properties of alkali-activated fly ash geopolymers. J. Hazard. Mater..

[36] Dai X., Aydin S., Yardimci M.Y., Lesage K., de Schutter G. (2020). Influence of water to binder ratio on the rheology and structural Build-up of Alkali-Activated Slag/Fly ash mixtures. Constr. Build. Mater..

[37] Lau C.K., Rowles M.R., Parnham G.N., Htut T., Ng T.S. (2019). Investigation of geopolymers containing fly ash and ground-granulated blast-furnace slag blended by amorphous ratios. Constr. Build. Mater..

[38] Zhang S., Li Z., Ghiassi B., Yin S., Ye G. (2021). Fracture properties and microstructure formation of hardened alkali-activated slag/fly ash pastes. Cem. Concr. Res..

[39] Wafa M.A., Fukuzawa K. (2014). Simultaneous effect of alkali activator and water/slag cement ratios on composites properties by full replacement of Portland cement. J. Compos. Mater..

[40] El-Wafa M.A., Fukuzawa K. (2018). Early-Age Strength of Alkali-Activated Municipal Slag–Fly Ash–Based Geopolymer Mortar. J. Mater. Civ. Eng..

[41] ACI (American Concrete Institute) (1997). Prediction of Creep, Shrinkage, and Temperature Effects in Concrete Structures.

[42] ACI (American Concrete Institute) (2011). Building Code Requirements for Structural Concrete.

[43] Wardhono A., Law D., Strano A. (2015). The Strength of Alkali-activated Slag/fly Ash Mortar Blends at Ambient Temperature. Procedia Eng..

[44] Sajedi F., Razak H.A., Bin Mahmud H., Shafigh P. (2012). Relationships between compressive strength of cement–slag mortars under air and water curing regimes. Constr. Build. Mater..

[45] Tossavainen M., Engstrom F., Yang Q., Menad N.-E., Larsson M.L., Bjorkman B. (2007). Characteristics of steel slag under different cooling conditions. Waste Manag..

[46] Pal S., Mukherjee A., Pathak S. (2003). Investigation of hydraulic activity of ground granulated blast furnace slag in concrete. Cem. Concr. Res..

[47] ASTM (American Society for Testing and Materials) (1978). Annual Book of ASTM Standards, Specification for Fly Ash and Raw or Calcined Natural Pozzolan for Use as a Mineral Admixture in Portland Cement Concrete.

[48] JSA (Japanese Standards Association) (2014). Method of Test for Slump Flow of Concrete.

[49] JSA (Japanese Standards Association) (2006). Method of Test for Compressive Strength of Concrete.

[50] JSA (Japanese Standards Association) (2006). Method of Test for Splitting Tensile Strength of Concrete.

[51] Yang K.-H., Cho A.-R., Song J.-K. (2012). Effect of water–binder ratio on the mechanical properties of calcium hydroxide-based alkali-activated slag concrete. Constr. Build. Mater..

[52] Chi M. (2012). Effects of dosage of alkali-activated solution and curing conditions on the properties and durability of alkali-activated slag concrete. Constr. Build. Mater..

[53] Yang K.-H., Song J.-K., Ashour A.F., Lee E.-T. (2008). Properties of cementless mortars activated by sodium silicate. Constr. Build. Mater..

[54] Fang G., Ho W.K., Tu W., Zhang M. (2018). Workability and mechanical properties of alkali-activated fly ash-slag concrete cured at ambient temperature. Constr. Build. Mater..

